# Longitudinal Evaluation of Humoral and Cellular Immunity After BNT162b2 COVID-19 Vaccination: Influence of Booster Type, Infection and Chronic Health Conditions

**DOI:** 10.3390/vaccines13101031

**Published:** 2025-10-02

**Authors:** Chiara Orlandi, Ilaria Conti, Davide Torre, Simone Barocci, Mauro Magnani, Giuseppe Stefanetti, Anna Casabianca

**Affiliations:** 1 Department of Biomolecular Sciences, Section of Biochemistry and Biotechnology, University of Urbino Carlo Bo, Via Ca’ Le Suore 2, 61029 Urbino, PU, Italy; chiara.orlandi@uniurb.it (C.O.); ilaria.conti@uniurb.it (I.C.); d.torre1@campus.uniurb.it (D.T.); mauro.magnani@uniurb.it (M.M.); 2 Laboratorio Covid, University of Urbino Carlo Bo, Via Arco d’Augusto 2, 61032 Fano, PU, Italy; 3 Department of Clinical Pathology, Azienda Sanitaria Territoriale (AST) di Pesaro-Urbino, Viale Comandino 70, 61029 Urbino, PU, Italy; simone.barocci@sanita.marche.it

**Keywords:** SARS-CoV-2, COVID-19, BNT, chronic disease, antibody level, B-cell response, T-cell response, long-term immunity

## Abstract

**Background/Objectives:** Understanding the durability of immunity induced by mRNA COVID-19 vaccines, especially in individuals with chronic health conditions, remains essential for guiding booster strategies. We conducted a longitudinal study to evaluate humoral and cellular immune responses up to 21 months after a primary two-dose BNT162b2 vaccination followed by a booster, either homologous (BNT162b2) or heterologous (mRNA-1273). **Methods:** Twenty-eight adults, mostly with chronic conditions, were assessed at approximately 9, 12 and 21 months post-primary vaccination. Serum anti-trimeric Spike IgG levels were quantified, and peripheral blood mononuclear cells were analyzed at 21 months for Spike-specific memory B-cell and T-cell responses by flow cytometry. **Results:** Participants were stratified by booster type, prior SARS-CoV-2 infection and health status. Anti-Spike IgG persisted in all participants but declined over time. The heterologous mRNA-1273 booster induced higher antibody titers at 9 months, while the homologous BNT162b2 booster led to more sustained antibody levels and higher frequencies of Spike-specific memory B cells at 21 months. Prior infection significantly enhanced antibody titers, particularly in homologous booster recipients. Surprisingly, individuals with chronic health conditions exhibited equal or higher antibody levels compared to healthy participants at all time points. At 21 months, robust Spike-specific class-switched memory B cells and polyfunctional CD4^+^ and CD8^+^ T-cell responses were detected. **Conclusions:** These findings demonstrate that BNT162b2 vaccination elicits durable, multi-layered immunity lasting nearly two years, even in individuals with chronic conditions, and support the use of both homologous and heterologous mRNA boosters to sustain protection in diverse populations.

## 1. Introduction

The rapid spread of coronavirus disease 2019 [COVID-19] and its related mortality required the rapid development and introduction of different vaccination strategies to counteract its causative agent (severe acute respiratory syndrome coronavirus 2 [SARS-CoV-2]) [1]. Leading vaccine platforms included mRNA-based vaccines (BNT162b2 [BNT] by BioNTech/Pfizer and Spikevax [mRNA-1273] by Moderna [2,3]) and a chimpanzee-adenovirus vector-based vaccine (ChAdOx1-S-nCoV-19 [ChAd] by AstraZeneca [4]). These vaccines were administered broadly, including to vulnerable groups such as the elderly and those with chronic illnesses, under emergency use approval [5,6]. Owing to the pandemic’s urgency, heterologous prime–boost strategies (mixing vaccine types for primary and/or booster series) were also explored to enhance immunity and compensate for supply or safety issues [7,8,9]. However, few studies reported on long-term vaccine efficacy and immunogenicity up to 12 months, while data on further extended follow-ups were initially lacking [7,10,11]. Follow-up studies focused on immune system activation and protection against several SARS-CoV-2 variants of concern [VOCs] after COVID-19 vaccination [12,13]. However, regarding particular population categories, such as those affected by diabetes or cancer, research evaluated mostly side effects and acceptance rates of vaccination [14,15].

In a previous study, we evaluated humoral and cellular responses up to 21 months post-vaccination in a cohort receiving various COVID-19 vaccine schedules [16]. When comparing heterologous ChAd/BNT versus homologous ChAd/ChAd regimens over this extended follow-up, we found that ChAd/BNT conferred superior long-term antibody persistence and cellular immunity [16]. The present study homes in on the subgroup of individuals who received a homologous BNT/BNT primary series. We chose to analyze this group separately because a majority of these BNT/BNT vaccinees had underlying chronic pathologies (over 70% reported conditions such as cardiovascular or respiratory diseases, diabetes or autoimmune disorders). This offered a unique opportunity to investigate how chronic health status may affect long-term vaccine-induced immunity.

Here, we characterize the Spike-specific humoral and cellular immune responses in the BNT/BNT cohort up to 21 months after the initial vaccination. We stratified participants by health status (chronically ill vs. healthy) and by the type of booster received at ~9 months (homologous BNT vs. heterologous mRNA-1273), as well as by intervening SARS-CoV-2 infection, to determine their impacts on immunity longevity. We specifically measured anti-Spike IgG antibody levels over time and assessed memory B-cell and T-cell responses at the 21-month time point. Our aim was to discern whether long-term BNT-induced immunity is maintained or modulated in individuals with chronic illnesses and to compare booster strategies in extending protection.

## 2. Materials and Methods

### 2.1. Cohort Characteristics

**Figure 1:**
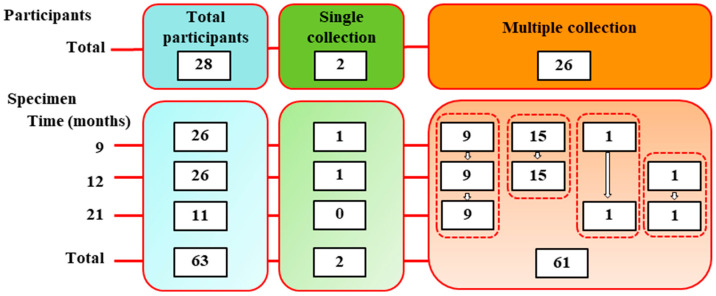
Flow diagram on participant recruitment and sample collection at each time point and across multiple time points throughout the study.

The subjects of this study (n = 28) were selected from a bigger cohort recruited among personnel from the Carlo Bo University of Urbino (Urbino, PU, Italy), as described previously [16,17,18]. All individuals received BNT/BNT priming from December 2020 to June 2021 and afterwards were vaccinated with a booster dose (BNT or mRNA-1273) from October 2021 to January 2022 (Figure 1, Table S1). Moreover, none declared a COVID-19 infection prior to vaccination. Participant demographic characteristics (sex, age, and body mass index [BMI]) by booster group are summarized in Table 1 and Table S2. Most participants were young to middle-aged adults, and 71% (20/28) reported having at least one chronic underlying medical condition (e.g., diabetes, cardiovascular or respiratory disease, autoimmune disorder, etc.) not related to COVID-19.

Blood samples were collected at 9 (n = 26), 12 (n = 26) and 21 months (n = 11) after the primary vaccination (equivalent to 3, 6 and 15 month post-booster) for serological analysis (Figure 1, Table S1). A subset of participants (n = 11) at the ~21-month time point was further screened for cellular immune assays (memory B-cell and T-cell analysis). Of the 28 participants, 9 individuals completed all three scheduled blood draws; 17 individuals provided samples at two time points; and 2 individuals gave a sample at only one time point during the study (Figure 1). Participants were also classified by SARS-CoV-2 infection status over the study period. At each visit, an anti-nucleocapsid (anti-N) IgG/IgM ELISA was performed to detect prior SARS-CoV-2 infection. Individuals testing positive for anti-N antibodies at any time were categorized as N+, whereas those who were negative for anti-N throughout were categorized as N− (Figure S1). Additionally, participants were categorized by health status: those who self-reported one or more chronic health conditions were labeled “chronically ill”, and those reporting no chronic conditions were labeled “healthy”. Chronic pathologies in this cohort included cardiovascular disease, diabetes, respiratory illness (e.g., asthma or COPD), autoimmune thyroiditis, multiple sclerosis and others (Figure S2, Table S3); severity and treatment status were not considered in this study.

### 2.2. Blood Collection and Processing

Clear serum from whole blood samples collected at the Laboratory of Clinical Pathology of Urbino Hospital (AST Azienda Sanitaria Territoriale Pesaro—Urbino) was obtained using serum separator tubes (SST) by centrifugation at 1500× *g* for 10 min following at initial clotting for 30 min at room temperature. Serum aliquots were immediately frozen at −80 °C until analysis.

For the isolation of PBMC, EDTA-collected whole blood was processed using Lymphoprep™ density gradient medium. The obtained PBMCs were cultured overnight at 37 °C and 5% CO_2_ in humified atmosphere in Roswell Park Memorial Institute 1640 [RPMI-1640] complete medium supplemented with 10% fetal bovine serum [FBS], 2 mM L-glutamine, 25 mM HEPES and 1% penicillin-streptomycin. Alternatively, PBMCs were cryopreserved in FBS supplemented with 10% dimethyl sulfoxide [DMSO] at −80 °C for further analyses.

### 2.3. Evaluation of Anti-SARS-CoV-2 Antibody Levels

The LIAISON^®^ SARS-CoV-2 TrimericS IgG chemiluminescent immunoassay (CLIA) was used to quantify serum IgG antibodies specific to the SARS-CoV-2 Spike protein (trimeric S) as previously described [16,17]. This assay has high specificity (99.5%) and sensitivity (98.7%) and demonstrates a strong negative (96.9%) and positive (100.0%) percent agreement when compared with neutralizing IgG antibodies. Results are reported in binding antibody units per milliliter (BAU/mL), using a specified conversion factor of 2.6 (BAU/mL = AU/mL × 2.6). The range of quantification was between 4.81 and 2080 BAU/mL and the cut-off for positivity was ≥33.8 BAU/mL [19,20]. For over-range samples (upper 2080 BAU/mL), sera were appropriately diluted 1:20 or 1:5 using the LIAISON^®^ TrimericS IgG Diluent Accessory according to the manufacturer’s recommendations and re-tested to obtain quantitative values. Samples were measured in a single well and the standard reference curve was included in each run. Each sample was also tested for antibodies against the SARS-CoV-2 nucleocapsid (N) protein using both COVID-19 ELISA IgM and COVID-19 ELISA IgG kits (Diatheva srl, Cartoceto, PU, Italy), according to the specific instructions provided by the manufacturer. Each sample was measured in duplicate and each run included negative control as duplicate, positive control as single test and sample diluent Buffer A as blank as duplicate. The anti-N results were used to classify participants as N+ or N−.

### 2.4. T-Cell Activation and Intracellular Cytokine Assays

The SARS-CoV-2 Prot_S T Cell Analysis Kit (Miltenyi Biotec B.V. & Co. KG, Bergisch Gladbach; Germany) was used to evaluate SARS-CoV-2-specific T-cell responses. Isolated PBMCs (1 × 10^6^), thawed or fresh, were rested at 37 °C overnight and then cultured in 96-well plates with a pool of 15-mer peptides overlapping by 11 amino acids covering the complete coding sequence (amino acids 5–1273) of the SARS-CoV-2 spike (S) glycoprotein (GenBank MN908947.3, protein QHD43416.1), supplied as PepTivator^®^ SARS-CoV-2 Prot_S Complete, premium grade (Miltenyi Biotech). After 2 h of stimulation at 37 °C, Brefeldin A (1 μg/mL) was added to inhibit cytokine secretion, and incubation proceeded for a total of 6 h.

After stimulation, the samples were washed with phosphate buffer containing 0.5% bovine serum albumin (BSA) and 2 mM EDTA [PEB buffer]. The cells were then fixed and permeabilized using the Inside fix and Inside Perm kit solutions followed by 20 min incubation with an antibody cocktail including CD3-APC, CD4-Vio^®^ Bright B515, CD8-VioGreen™, CD20-VioBlue^®^, CD154-APC-Vio770, IFN-γ-PE and TNF-α-PE-Vio770. Viability 405/452 Fixable Dye was used for discrimination between live and dead cells.

BD FACS Canto II flow cytometer was used for sample acquisition. The gating strategy applied to identify intracellular cytokine production and activation markers in CD4^+^ and CD8^+^ T-cell populations is shown in Figure S3 and was previously described [16].

### 2.5. Analysis of SARS-CoV-2-Specific B Cells

The SARS-CoV-2 Spike B Cell Analysis Kit was used to analyzespecific Spike antibodies expressed by human B-cells (Miltenyi Biotec B.V. & Co. KG, Bergisch Gladbach; Germany). PBMCs (5–10 × 10^6^), thawed or fresh were rested at 37 °C overnight in complete RPMI-1640 medium. After incubation, PBMCs were washed in PEB buffer and stained at +4 °C for 30 min with an antibody mixture containing the following reagents: Recombinant SARS-CoV-2 Spike-Protein (HEK)-Biotin-Streptavidin-PE-Vio^®^ 770, Recombinant SARS-CoV-2 Spike-Protein (HEK)-Biotin-Streptavidin-PE, CD27-Vio Bright FITC IgG-VioBlue^®^, CD19-APC-Vio^®^ 770 and IgM-APC. 7-AAD staining was used to discriminate live/dead cells.

BD FACS Canto II flow cytometer was used for sample acquisition. The gating strategy applied to identify SARS-CoV-2-specific memory B cells is shown in Figure S4 and was previously described [16].

### 2.6. Statistical Analysis

The Mann–Whitney U test was employed to compare IgG levels between two independent vaccination groups. The Kruskal–Wallis test followed by Dunn’s multiple comparisons post-test for repeated measures was applied within the same group across different time points.

The relationship between demographic or clinical variables and vaccination groups was estimated using the chi-squared (χ^2^) test for categorical variables and the Kruskal–Wallis test followed by Dunn’s multiple comparisons post-test for continuous variables. Relationship among clinical parameters and vaccination schedule was analyzed by χ^2^.

For the analysis of cellular responses, the Mann–Whitney U test was employed for comparisons between two independent groups.

*p*-values < 0.05 were considered statistically significant. All analyses and visualizations were conducted using GraphPad Prism version 8.4.2 (GraphPad Software, San Diego, CA, USA). Statistical tests used for specific comparisons are described in figure legends.

## 3. Results

### 3.1. SARS-CoV-2 Longitudinal Analysis of Anti-Trimeric Spike IgG Levels at 9, 12 and 21 Months After Vaccination

**Figure 2:**
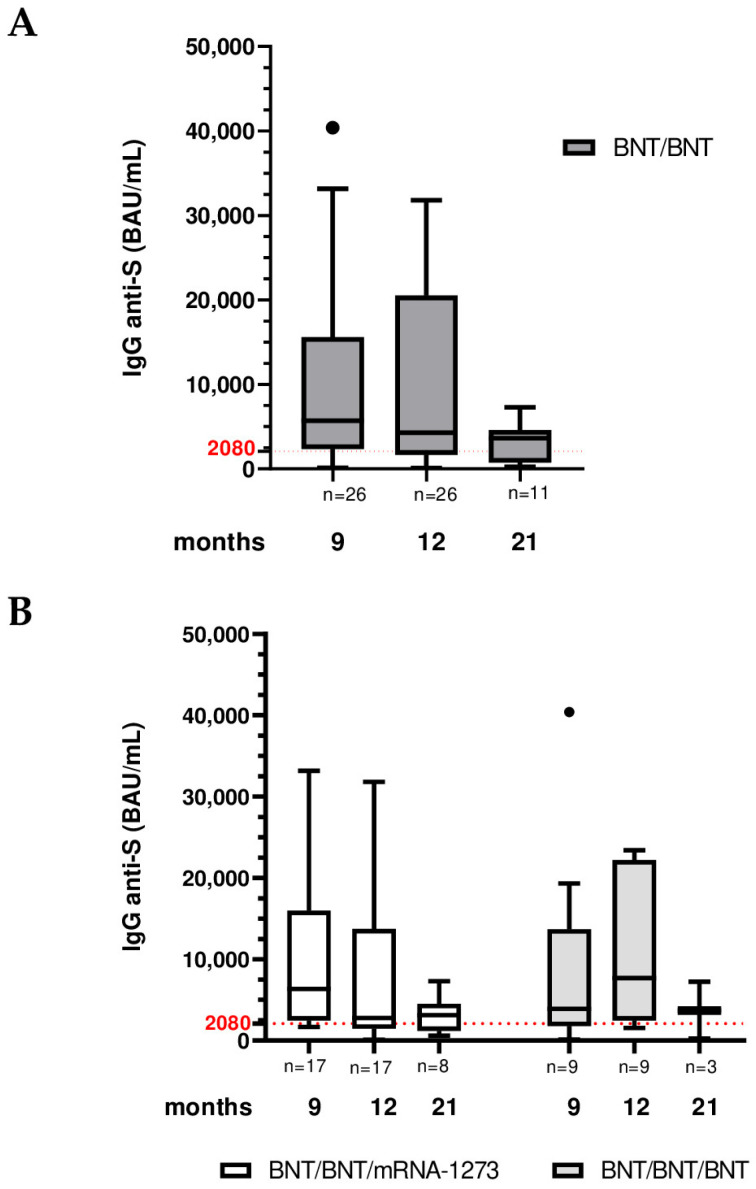
Comparison of SARS-CoV-2 anti-trimeric Spike protein IgG levels of vaccinated subjects at 9, 12 and 21 months after BNT/BNT vaccination. (**A**) IgG levels across all BNT/BNT vaccinated subjects. (**B**) IgG levels across BNT/BNT/mRNA-1273 and BNT/BNT/BNT vaccinated subjects. Numbers of patients are reported in the figures. Boxplots display the interquartile range [IQR] and median, with the lowest and highest values represented by whiskers (Tukey-style). Outliers are showed as distinct points. Pairwise Mann–Whitney test and Kruskal–Wallis test followed by Dunn multiple comparison post hoc.

All participants maintained a positive response to anti-trimeric Spike IgG throughout the 21-month follow-up. When considering the cohort as a whole, anti-S IgG titers declined over time from 9 to 21 months post-vaccination (Table 2, Figure 2A). The median anti-Spike IgG concentration across all BNT/BNT vaccinees was 5700 BAU/mL at 9 months, which fell to 4280 BAU/mL at 12 months and further to 3640 BAU/mL by 21 months (Table 2).

However, antibody kinetics differed markedly by booster regimen. In the heterologous booster (BNT/BNT/mRNA-1273) group, median IgG titers dropped from 6340 BAU/mL at 9 months to 2780 BAU/mL at 12 months, then showed a slight increase to 3130 BAU/mL at 21 months (Table 2). In contrast, the homologous booster (BNT/BNT/BNT) group exhibited a transient antibody rise at the 12-month time point: median IgG climbed from 3900 BAU/mL at 9 months to 7680 BAU/mL at 12 months, then declined to 3680 BAU/mL by 21 months (Table 2, Figure 2B). Inter-group comparisons revealed that, despite not reaching statistical significance in pairwise tests, the BNT/BNT/mRNA-1273 regimen elicited a higher anti-Spike IgG levels at 9 months post-primary vaccination compared to the BNT/BNT/BNT schedule; conversely at the 12-month time point a higher anti-Spike IgG levels was observed in the BNT/BNT/BNT group (2780 BAU/mL for BNT/BNT/mRNA-1273 and 7680 BAU/mL for BNT/BNT/BNT). Then both groups maintained comparable IgG levels at 21 months (Table 2, Figure 2B).

### 3.2. Role of SARS-CoV-2 Infection on Humoral Immunity

To evaluate viral infection and its impact on humoral immunity, anti-trimeric Spike IgG levels were compared, grouping participants according to their infection status. By 21 months, a large proportion of the cohort had experienced SARS-CoV-2 infection as indicated by anti-nucleocapsid seroconversion: the percentage of N+ participants increased from ~30% at 9 months to >70% at 21 months, with similar infection rates in both booster groups (heterologous and homologous) at each time point (Figure S1).

Importantly, prior SARS-CoV-2 infection (N+) was associated with higher Spike IgG levels compared to no infection (N−) at all matching time points. For example, at 12 months, the median anti-S IgG titer in N+ individuals was ~7680 BAU/mL vs. 2780 BAU/mL in N− individuals (when considering all BNT/BNT participants, regardless of booster; *p* = 0.1484).

**Figure 3:**
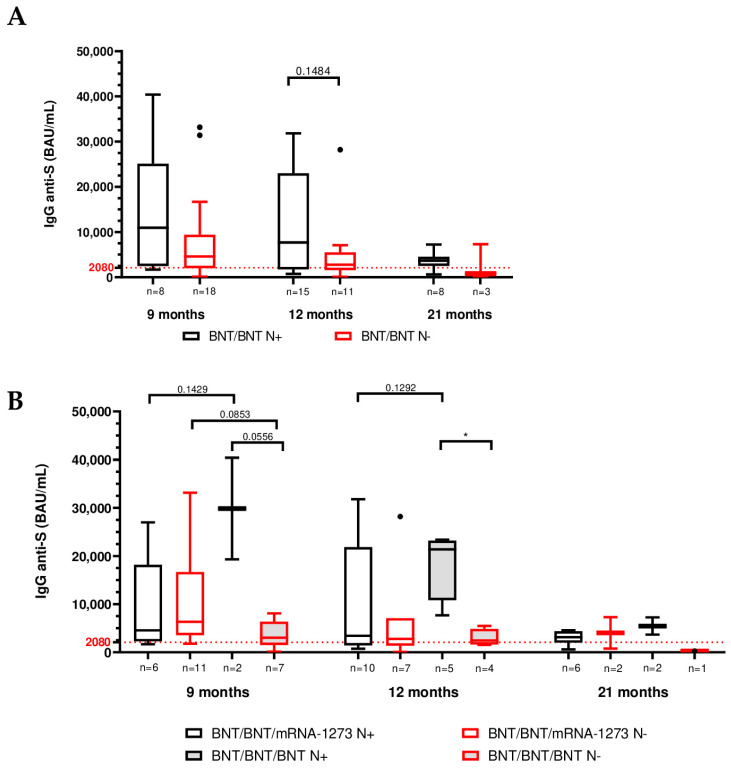
Inter-group comparison of SARS-CoV-2 anti-trimeric Spike protein IgG levels in BNT/BNT-vaccinated subjects at 9, 12 and 21 months after vaccination stratified by infection status (N+ = infected, N− = uninfected). (**A**) Comparison of IgG titers across all BNT/BNT subjects. (**B**) Comparison of IgG titers across BNT/BNT/mRNA-1273- and BNT/BNT/BNT-vaccinated subjects. Numbers of patients are reported in the figures. Boxplots display the interquartile range [IQR] and median, with the lowest and highest values represented by whiskers (Tukey-style). Outliers are showed as distinct points. Pairwise Mann–Whitney test and Kruskal–Wallis test followed by Dunn multiple comparison post hoc. * *p* < 0.05.

Inter-group comparison between the two vaccination schedules showed highest anti-S antibody titers for the BNT/BNT/BNT N+ individuals across the study (median value: 29,860, 21,400 and 5465 at 9-, 12- and 21-month follow-up) which particularly differed from BNT/BNT/mRNA-1273 N+ subjects at both months 9 and 12, although without statistical significance (*p* value = 0.1429 and 0.1292, respectively) (Table 3, Figure 3B). Conversely among N− individuals, those immunized with BNT/BNT/BNT displayed lower anti-Spike IgG titers compared to the BNT/BNT/mRNA-1273 group across all time points, particularly at 9 months (*p* value = 0.0853) and 21 months (although only three subjects were compared) (Table 3, Figure 3B). Moreover, intra-group comparison showed higher antibody levels for N+ BNT/BNT/BNT-vaccinated subjects compared to N− participants, with statistically significant differences at month 12 (*p* value = 0.0556) (Table 3, Figure 3B). By contrast, in the heterologous BNT/BNT/mRNA-1273 group, the pattern was different: N− individuals had comparable (12 months) or slightly higher (9 and 21 months) IgG titers than N+ individuals (Table 3, Figure 3B).

### 3.3. Clinical Characteristics of the Study Groups at 9, 12 and 21 Months Post-Vaccination

We documented the prevalence of chronic health conditions (“chronically ill” status) in the cohort over time. An increasing percentage of participants reported chronic pathologies from 9 to 21 months post-vaccination (from 69.2% at 9 months to 81.8% at 21 months; Figure S2). Chronic conditions encompassed a range of illnesses as noted earlier, including metabolic, cardiovascular, respiratory and autoimmune diseases (Table S3). No single disease was shared by all participants, though diabetes was the most common single condition, present in several individuals across groups. When comparing the two booster groups, we found that the heterologous BNT/BNT/mRNA-1273 group consistently had a higher proportion of chronically ill participants than the homologous BNT/BNT/BNT group at each time point. Specifically, in the heterologous group 76.5% of participants reported health conditions at both 9 and 12 months, rising to 87.5% by 21 months, whereas in the homologous group, 55.6% reported health conditions at 9 months and 66.7% at 12 and 21 months (Figure S5).

### 3.4. Impact of Chronic Health Status on Humoral Immunity

**Figure 4:**
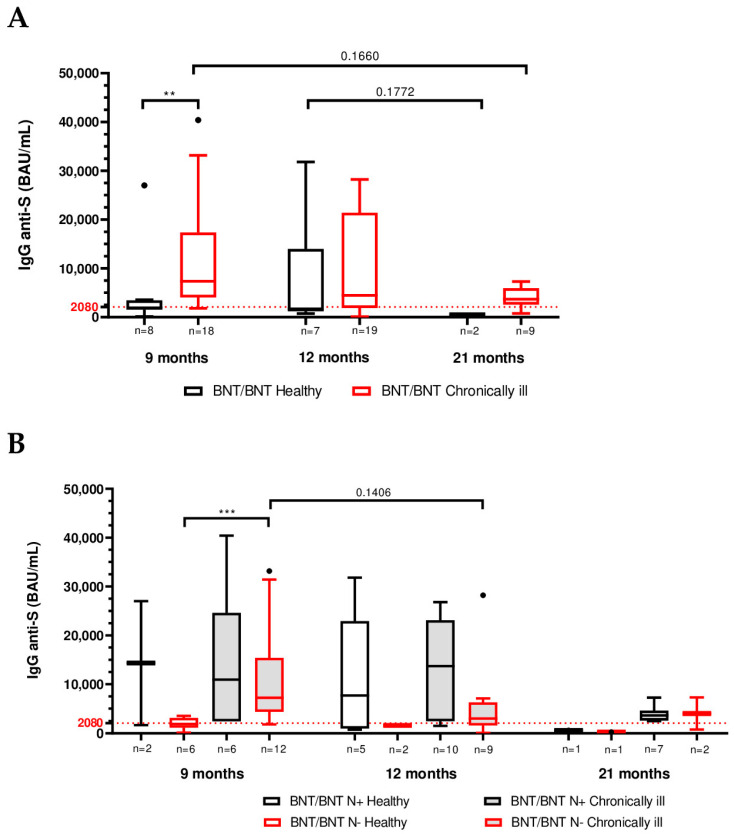
Comparison of SARS-CoV-2 anti-trimeric Spike protein IgG in BNT/BNT-vaccinated subjects at 9, 12 and 21 months after vaccination stratified by both infection status (N+ = infected; N− = uninfected) and health status (healthy and chronically ill). (**A**) Comparison of IgG titers between healthy or chronically ill subjects. (**B**) Comparison of IgG titers between subjects positive/negative to previous viral infection (i.e., N+/N−) and healthy or chronically ill. Number of patients reported in the figure. Boxplots display the interquartile range [IQR] and median, with the lowest and highest values represented by whiskers (Tukey-style). Outliers are shown as distinct points. Pairwise Mann–Whitney test and Kruskal–Wallis test followed by Dunn multiple comparison post hoc. ** *p* < 0.01; *** *p* < 0.001.

To determine if having a chronic medical condition influenced vaccine antibody responses, we compared anti-Spike IgG levels between “healthy” and “chronically ill” participants at each time point. Strikingly, chronically ill individuals had equal or higher IgG titers compared to healthy individuals at all measured time points. In particular, at 9 months, the chronically ill group’s IgG levels were significantly higher than those of healthy participants (7370 vs. 1915 BAU/mL; *p* < 0.01, Figure 4A and Table 4). By 12 and 21 months, chronically ill participants continued to show higher median antibody titers than healthy participants (Figure 4A, Table 4), although the differences at those later points did not reach statistical significance (*p* > 0.1) likely due to wider variability and smaller “healthy” sample sizes in the later follow-ups.

We next examined each booster group separately. In the homologous BNT/BNT/BNT group, the chronically ill group showed higher IgG titers than their healthy counterparts at all time points, with the difference at 9 months being statistically significant (8080 vs. 1780 BAU/mL, *p* < 0.05; Figure S6). In the heterologous BNT/BNT/mRNA-1273 group, chronically ill participants also displayed higher IgG titers than healthy at 9, 12 and 21 months (at 9 months: 6660 vs. 2680 BAU/mL; at 12 months: 4100 vs. 1453 BAU/mL; and at 21 months: 3640 vs. 618 BAU/mL median), although these differences did not reach significance, likely also due to the small number of healthy individuals in that group (Figure S6).

Furthermore, when infection status was also considered, the trend of higher IgG titers in individuals with chronic conditions remained evident. A general decrease on anti-S antibody levels was observed from month 9 to 21 for all the subgroups with the exception for N− chronically ill participants, who showed a major, although not statistically significant, decrease from month 9 (7230 BAU/mL) to 12 (3040 BAU/mL), *p*-value = 0.1406 (Table 5). Following inter-group comparison, N+ participants (both healthy and chronically ill) showed higher anti-Spike IgG titers compared to the respective N− subgroup across all the study, although with not statistically significant differences (Table 5, Figure 4B). However, statistically significant higher anti-S IgG titer was evidenced in N− chronically ill subjects compared to N− healthy individuals at 9 months (*p* < 0.001) (Figure 4B).

Further stratification of participants according to both vaccination schedule and infection status other than healthy status showed a common trend of higher anti-S IgG titer for N+ individuals compared to the respective N− group at all the time points of the study (both in healthy and chronically ill subjects and in the two vaccination groups), except for BNT/BNT/mRNA-1273 chronically ill participants at month 9, in which the antibody median value of the N− individuals was higher than that of N+ subjects (8700 vs. 4570 BAU/mL). Across the study period, anti-Spike IgG levels declined from month 9 to 21 in all stratified groups. Notably, the decline was less pronounced between months 9 and 12 in N+ individuals with chronic conditions. For example, in the BNT/BNT/mRNA-1273 group, median IgG titers in N+ individuals with chronic conditions were 4570, 4100 and 3640 BAU/mL at months 9, 12, and 21, respectively. In the BNT/BNT/BNT group, the same subgroup showed median titers of 29,860, 23,000 and 5465 BAU/mL at the corresponding time points. Throughout the entire follow-up period, the BNT/BNT/BNT N+ subgroup with chronic conditions consistently displayed the highest anti-Spike IgG titers (Table S4).

### 3.5. Spike-Specific Cellular Response at 21 Months Post-Vaccination

To evaluate long-term cellular immunity, we analyzed Spike-specific memory B cells and T-cell induction in participant PBMCs at the final 21-month time point. Despite the small sample available for these assays, clear antigen-specific memory B-cell responses were detectable in both booster groups (Figure S7). Spike-binding memory B cells were identified and further characterized by isotype as IgG^+^ (class-switched) or IgM^+^ (unswitched). We observed that IgG^+^ memory B cells (IgM^−^/IgG^+^ MBCs) greatly outnumbered IgM^+^ memory B cells in all subjects, consistent with a robust class-switched memory imprint from vaccination. This indicates that the majority of Spike-specific memory B cells had undergone isotype switching to IgG, a hallmark of a mature immune memory response.

When comparing booster strategies, the homologous BNT/BNT/BNT group showed a trend toward higher frequencies of Spike-specific memory B cells (both IgG^+^ and IgM^+^ subsets) than the heterologous group, although likely due to limited sample size these differences were not statistically significant.

At 21 months post-vaccination, Spike-specific IgG^+^ memory B cells were detected in N+ individuals, whereas only minimal levels were observed in the single uninfected (N−) participant (Figure S8). Due to the limited sample size of the N− group (n = 1), no conclusions can be drawn regarding differences between groups.

Regarding T-cell activation, only BNT/BNT/mRNA-1273 samples were analyzed. Expression of IFNγ, TNFα and CD154 in CD4^+^ T cells, as well as IFNγ and TNFα in CD8^+^ T cells, was observed in N+ individuals. Limited data were available for N− participants (n = 1), precluding meaningful comparison between groups (Figure S9A,B).

## 4. Discussion

Since the SARS-CoV-2 emergency, vaccination strategies—particularly novel mRNA-based vaccines—have drastically reduced COVID-19-associated morbidity and mortality globally [3,21]. Nevertheless, with continued viral evolution and emergence of variants that can evade immune responses, evaluating long-term vaccine-induced immunity remains critical [7,8,22,23,24,25]. Recently, our research group extensively studied humoral and cellular immune responses elicited by different COVID-19 vaccination schedules, assessing their persistence up to 21 months after primary vaccination [16,17,18]. These studies demonstrated superior immune durability following heterologous vaccination (ChAd/BNT162b2, ChAd/BNT) compared to homologous regimens (ChAd/ChAd, BNT/BNT). Specifically, ChAd/BNT recipients showed significantly higher anti-Spike (S) IgG titers at two months post-primary immunization [17], sustained elevated antibody levels at six months [18] and prolonged antibody persistence compared to homologous regimens [16]. At 21 months, ChAd/BNT vaccination also elicited enhanced memory B-cell responses (IgM^−^/IgG^+^ phenotype) and stronger CD8^+^ T-cell cytokine production, especially in individuals with hybrid immunity from prior infection [16]. Building upon this foundation, the present study specifically addressed the durability of humoral and cellular immune responses after homologous (BNT) or heterologous (mRNA-1273) mRNA booster administration following a primary BNT vaccination, focusing on chronic health conditions and intervening SARS-CoV-2 infections as modulatory factors. Sustained anti-Spike IgG antibodies, memory B-cell and T-cell responses persisted despite gradual declines over time. Importantly, robust immune responses were consistently observed even among chronically ill individuals, highlighting the lasting protective efficacy of mRNA vaccines in these vulnerable populations.

We observed distinct antibody kinetics according to booster type. Subjects receiving a heterologous mRNA-1273 booster showed initially higher anti-Spike IgG levels at 9 months, consistent with earlier evidence supporting immunological benefits of mixing vaccine platforms [25,26]. However, homologous BNT booster induced more sustained antibody responses over the longer term (12–21 months), aligning with other recent studies suggesting differing advantages between heterologous (short-term robustness) and homologous (long-term persistence) boosters [12,16,23,27].

The occurrence of SARS-CoV-2 infection during follow-up notably influenced antibody levels, acting as a strong natural booster, especially evident in recipients of homologous BNT boosters. This observation supports existing data demonstrating superior antibody responses in individuals with hybrid immunity, generated by vaccination and natural infection, compared to vaccination alone [16,28,29]. Despite widespread Omicron-driven infections, severe disease was not observed, underscoring sustained clinical protection conferred by vaccine-induced immunity.

Another important finding relates to individuals with chronic health conditions. Contrary to expectations that chronic illness might impair vaccine-induced immunity [30,31,32], chronically ill subjects consistently exhibited equal or higher antibody responses compared to healthy controls at all evaluated time points. For example, at 9 months, chronically ill subjects displayed significantly higher IgG titers, a trend persisting among nucleocapsid-negative (uninfected) participants. Recent reports similarly observed strong vaccine-induced antibody responses in patients with chronic diseases such as diabetes or obesity [33,34,35]. Although the exact mechanisms are unclear, heightened basal inflammatory states or altered immune regulation in chronic diseases might amplify responses to vaccination [36].

We further characterized cellular immunity at 21 months post-vaccination, revealing durable Spike-specific memory B-cell and T-cell responses irrespective of booster type, infection status, or chronic conditions. Predominance of class-switched (IgG^+^) memory B cells and sustained polyfunctional Spike-specific CD4^+^ and CD8^+^ T-cell responses underscore long-term protection despite declining antibody levels, consistent with prior evidence emphasizing T-cell-mediated immunity as critical for preventing severe disease [37,38].

Limitations of our study include modest sample size and participant attrition (n = 28 participants and only 11 subjects completed the follow-up), reducing statistical power particularly when stratifying by booster regimen, infection history and chronic conditions. Additionally, intermittent nucleocapsid serology may underestimate transient or seronegative infections. Grouping all chronic conditions together limited our ability to discern disease-specific effects on immune responses or medication impacts. Direct evidence of viral protection (e.g., viral neutralization assay) is lacking, and other clinical parameters (e.g., age, sex and BMI) were not further explore potentially restricting our results interpretation. Finally, lack of human leukocyte antigen [HLA] distribution within the study cohort could limit the interpretability and impact of our findings. Actually, HLA has an essential role in inducing an effective immune response and its heterogeneous composition (i.e., homozygous or heterozygous HLA class I and II variants) has been correlated to disease risk and infection susceptibility, as well as to the development of a protective immune response after vaccination [39,40,41,42].

## 5. Conclusions

To our knowledge, this is the first study conducted in Italy, with a predominantly young-to-middle-aged adult population enriched for chronic health conditions, to evaluate SARS-CoV-2–specific humoral and cellular immunity up to 21 months following BNT162b2 primary vaccination and booster doses. Our findings reinforce the durability and robustness of humoral and cellular immunity elicited by mRNA vaccination and booster strategies up to nearly two years post-primary vaccination, even in populations with chronic illnesses. Both homologous and heterologous boosters confer immunological advantages depending on the timing and immune parameters evaluated. Additionally, natural SARS-CoV-2 infection significantly enhances antibody responses induced by the vaccine, highlighting the potency of hybrid immunity. These results support current booster strategies and provide reassuring evidence for protecting vulnerable groups. Larger longitudinal studies exploring specific chronic conditions will further refine optimal vaccination approaches for at-risk populations.

## Figures and Tables

**Table 1:** Demographic characteristics of participants.

Vaccine Schedule				9 Months	12 Months	21 Months
Primary	Booster					
**BNT/BNT**	Total			n = 26	n = 26	n = 11
		Gender	Male	8/26 (30.8%)	8/26 (30.8%)	3/11 (27.3%)
		Age	Years, median (IQR)	54.5 (43.75–59.25)	53 (43.75–59.25)	53 (42–58)
		BMI	Median (IQR)	25.19 (22.8–28.74)	24.84 (22.18–28.74)	25.39 (22.41–28.69)
	mRNA-1273			n = 17	n = 17	n = 8
		Gender	Male	7/17 (41.2%)	7/17 (41.2%)	3/8 (37.5%)
		Age	Years, median (IQR)	57 (43.5–59.5)	56 (42.5–59.5)	56.5 (38.25–58)
		BMI	Median (IQR)	26.99 (24.62–28.9)	25.39 (24.03–28.9)	27.62 (25.38–28.94)
	BNT			n = 9	n = 9	n = 3
		Gender	Male	1/9 (11.1%)	1/9 (11.1%)	0/3 (0.0%)
		Age	Years, median (IQR)	50 (42–60.5)	53 (46.5–60.5)	50 (47–53)
		BMI	Median (IQR)	23.24 (20.79–26.67)	23.24 (20.88–26.67)	22.41 (19.49–23.24)

Number of participants grouped according to vaccine schedule is reported alongside with gender, age and body mass index [BMI]. BNT/BNT refers to BNT162b2 COVID-19 vaccine [BNT] administered at both first and second dose followed by BNT or Moderna [mRNA-1273] vaccination as third dose.

**Table 2:** SARS-CoV-2 anti-trimeric Spike IgG titers by vaccination schedule and time points.

		Total (BNT/BNT)	BNT/BNT/ mRNA-1273	BNT/BNT/ BNT
**IgG titer** **(BAU/mL)**	9 months Median (IQR)	n = 26 5700 (2358–15,600)	n = 17 (65.4%) 6340 (2470–15,960)	n = 9 (34.6%) 3900 (1780–13,700)
	12 months Median (IQR)	n = 26 4280 (1660–20,500)	n = 17 (65.4%) 2780 (1455–13,710)	n = 9 (34.6%) 7680 (2462–22,200)
	21 months Median (IQR)	n = 11 3640 (770–4600)	n = 8 (72.7%) 3130 (1198–4524)	n = 3 (27.3%) 3680 (256–7250)

SARS-CoV-2 anti-trimeric Spike IgG levels at 9, 12 and 21 months post-primary vaccination between two different groups of vaccinated subjects. Sample size (n) for each group and relative percentages are reported at each time point.

**Table 3:** SARS-CoV-2 anti-trimeric Spike IgG titers by vaccination schedule, time point and infection status.

		Total (BNT/BNT)		BNT/BNT/mRNA-1273		BNT/BNT/BNT	
		N+	N−	N+	N−	N+	N−
**IgG titer** **(BAU/mL)**	9 months	n = 8 (30.8%)	n = 18 (69.2%)	n = 6 (23.1%)	n = 11 (42.3%)	n = 2 (7.7%)	n = 7 (26.9%)
	Median	10,950	4580	4570	6340	29,860	3040
	(IQR)	(2465–25,080)	(1991–9390)	(2265–18,180)	(3580–16,680)	(19,320–40,400)	(1510–6380)
	12 months	n = 15 (57.7%)	n = 11 (42.3%)	n = 10 (38.5%)	n = 7 (26.9%)	n = 5 (19.2%)	n = 4 (15.4%)
	Median	7680	2780	3420	2780	21400	2462
	(IQR)	(1720–23,000)	(1540–5480)	(1449–21,850)	(1380–7100)	(10,830–23,200)	(1626–4870)
	21 months	n = 8 (72.7%)	n = 3 (27.3%)	n = 6 (54.5%)	n = 2 (18.2%)	n = 2 (18.2%)	n = 1 (9.1%)
	Median	3660	770	3130	4035	5465	256
	(IQR)	(2515–4524)	(256–7300)	(2015–4371)	(770–7300)	(3680–7250)	

SARS-CoV-2 anti-trimeric Spike IgG levels at 9, 12 and 21 months post-primary vaccination between two different groups of vaccinated subjects, stratified by infection status (N+ = infected, N− = uninfected). Sample size (n) for each group and relative percentages are reported at each time point. Data are presented as the number of subjects over the total within each subgroup, with relative percentages provided in parentheses.

**Table 4:** SARS-CoV-2 anti-trimeric Spike IgG titers by vaccination schedule, time point and health status.

		Total (BNT/BNT)		BNT/BNT/mRNA-1273		BNT/BNT/BNT	
		Healthy	Chronically Ill	Healthy	Chronically Ill	Healthy	Chronically Ill
**IgG titer** **(BAU/mL)**	9 months	n = 8 (30.8%)	n = 18 (69.2%)	n = 4 (23.5%)	n = 13 (76.5%)	n = 4 (44.4%)	n = 5 (55.6%)
	Median	1915	7370	2680	6660	1780	8080
	(IQR)	(1553–3445)	(4050–17,340)	(1705–21,145)	(3290–15,960)	(481–2793)	(5140–29,860)
	12 months	n = 7 (26.9%)	n = 19 (73.1%)	n = 4 (23.5%)	n = 13 (76.5%)	n = 3 (33.3%)	n = 6 (67.7%)
	Median	1700	4460	1453	4100	7680	13440
	(IQR)	(1206–13,980)	(1884–21,400)	(851–24,275)	(1625–13,710)	(1540–13,980)	(2751–23,100)
	21 months	n = 2 (18.2%)	n = 9 (81.8%)	n = 1 (12.5%)	n = 7 (87.5%)	n = 1 (33.3%)	n = 2 (66.7%)
	Median	437	3680	618	3640	256	5465
	(IQR)	(256–618)	(2550–5925)		(2480–4600)		(3680–7250)

SARS-CoV-2 anti-trimeric Spike IgG levels at 9, 12 and 21 months post-primary vaccination between two different groups of vaccinated subjects, stratified by health status (healthy or chronically ill). Sample size (n) for each group and relative percentages are reported at each time point.

**Table 5:** SARS-CoV-2 anti-trimeric Spike IgG titers by health status, infection status and time point.

		Healthy		Chronically Ill	
		N+	N−	N+	N−
**IgG titer** **(BAU/mL)**	9 months	n = 2 (7.7%)	n = 6 (23.1%)	n = 6 (23.1%)	n = 12 (46.2%)
	Median	14,340	1915	10,950	7230
	(IQR)	(1680–27,000)	(1167–3175)	(2475–24,590)	(4340–15,375)
	12 months	n = 5 (19.2%)	n = 2 (7.7%)	n = 10 (38.5%)	n = 9 (34.6%)
	Median	7680	1620	13710	3040
	(IQR)	(969.5–22,890)	(1540–1700)	(2485–23,100)	(1632–6290)
	21 months	n = 1 (9.1%)	n = 1 (9.1%)	n = 7 (63.6%)	n = 2 (18.2%)
	Median	618	256	3680	4035
	(IQR)			(2620–4600)	(770–7300)

SARS-CoV-2 anti-trimeric Spike IgG levels at 9, 12 and 21 months post-primary vaccination among the BNT/BNT vaccinated subjects, stratified by infection status and health status (healthy or chronically ill). Sample size (n) for each group and relative percentages are reported at each time point.

## Data Availability

The original contributions presented in this study are included in the article/Supplementary Materials. Further inquiries can be directed to the corresponding authors.

## Supplementary Materials

The following supporting information can be downloaded at https://www.mdpi.com/article/10.3390/vaccines13101031/s1: Figure S1: Percentages of SARS-CoV-2 infected/uninfected (i.e., N+ or N−, respectively) subjects vaccinated with two different administration schedules at 9, 12 and 21 months after vaccination. Figure S2: Percentages of healthy or chronically ill participants at 9, 12 and 21 months after BNT/BNT vaccination. Figure S3: Representative plots of the gating strategy used to identify SARS-CoV-2 specific T cell and quantify intracellular cytokine expression in CD4^+^ or CD8^+^. Figure S4: Representative plots of the gating strategy used to quantify the percentage of Spike-specific memory B-cell [MBCs] populations. Figure S5: Percentages of individuals with or without chronic health conditions between BNT/BNT/mRNA-1273 and BNT/BNT/BNT vaccinated groups at 9, 12 and 21 months after primary vaccination. Figure S6: Inter-group comparison of SARS-CoV-2 anti-trimeric Spike protein IgG between two different groups of healthy or chronically ill vaccinated subjects at 9, 12 and 21 months after vaccination. Figure S7: Inter-group comparison of the percentage of Spike-specific memory B cells [MBCs] between subjects receiving BNT/BNT/mRNA-1273 and BNT/BNT/BNT vaccination schedules at 21 months post-vaccination. Figure S8: Intra-group comparison of the percentage of Spike-specific memory B cells [MBCs] between infected/uninfected subjects receiving BNT/BNT vaccination schedules at 21 months post-vaccination. Figure S9: Intra-group comparison of the intracellular cytokine expression in CD4^+^ and CD8^+^ T-cell infected/uninfected BNT/BNT/mRNA-1273 vaccination groups at 21 months post-vaccination. Table S1: Timelines and booster schedules for recruited vaccinated subjects. Table S2: Relationship between demographic parameters and vaccination schedule. Table S3.: Infected [N+] or uninfected [N−] subjects vaccinated with BNT/BNT/mRNA-1273 or BNT/BNT/BNT, grouped according to their chronic condition at month 9, 12, 21. Table S4: Healthy and chronically ill subjects grouped according to SARS-CoV-2 infection (infected [N+] or uninfected [N−]) vaccinated with BNT/BNT/mRNA-1273 or BNT/BNT/BNT, at month 9, 12, 21.
