# Supplementary material for: Longitudinal Evaluation of Humoral and Cellular Immunity After BNT162b2 COVID-19 Vaccination: Influence of Booster Type, Infection and Chronic Health Conditions
Source: Vaccines (Basel). 2025 Oct 2;13(10):1031. doi: 10.3390/vaccines13101031 (PMC12567546; doi:10.3390/vaccines13101031)
Supplement: Supplementary file 1 [file vaccines-13-01031-s001.zip › vaccines-3822684-supplementary.pdf]

Supplementary Material

Table S1. Timelines and booster schedules for recruited vaccinated subjects.

Vaccine schedule			Follow-up after primary vaccination		
Primary		**Booster** (days, mean ± SD)	**Months 9** (days, mean ± SD)	**Months 12** (days, mean ± SD)	**Months 21** (days, mean ± SD)
All		213 ± 32	294 ± 37	388 ± 42	648 ± 43
BNT/BNT	mRNA-1273	214 ± 33	297 ± 30	392 ± 42	652 ± 42
	BNT	213 ± 33	288 ± 48	379 ± 43	636 ± 54

BNT/BNT refers to the BNT162b2 COVID-19 vaccine [BNT] administered as both the first and second doses (primary immunization). Within brackets, days described as mean ± standard deviation [SD], represent days after primary BNT/BNT immunization.

Table S2. Relationship between demographic parameters and vaccination schedule.

Variable	Vaccine Schedule		p value
	**BNT/BNT/mRNA-1273**	**BNT/BNT/BNT**	
Gender Male/Female	7/11	1/9	0.1049
Age median (IQR)	56.5 (29.0-65.0)	51.5 (35.0-70.0)	0.9341
BMI median (IQR)	26.2 (18.2-39.5)	22.8 (19.5-30.1)	0.0717

Chi-square (male/female) and nonparametric t tests (age and body mass index [BMI]) on demographic and clinical parameters related to the different vaccination groups. Significant p-value is considered below 0.05.

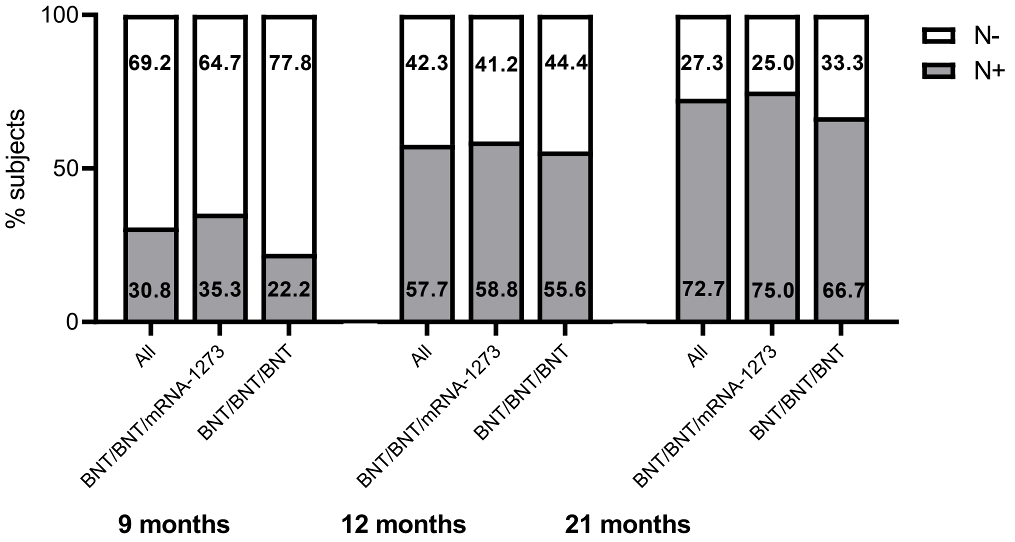

Figure S1. Percentages of SARS-CoV-2 infected/uninfected (i.e. N+ or N-, respectively) subjects vaccinated with two different administration schedules at 9, 12, and 21 months after vaccination.

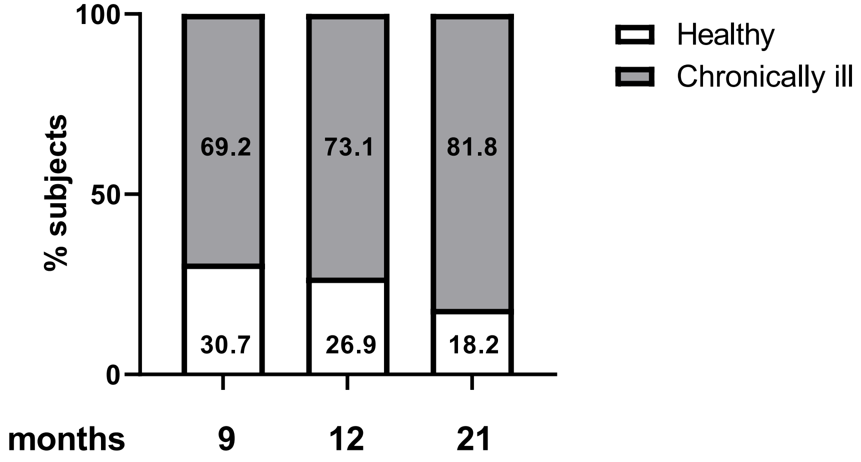

Figure S2. Percentages of healthy or chronically ill participants at 9, 12 and 21 months after BNT/BNT vaccination.

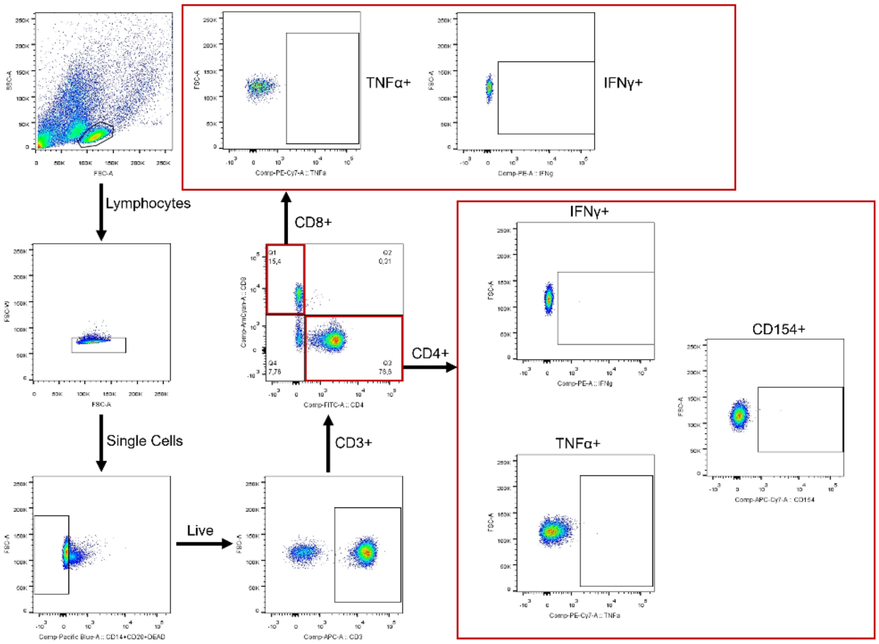

Figure S3. Representative plots (shown as pseudocolor) of the gating strategy used to identify SARS-CoV-2 specific T cell and quantify intracellular cytokine expression in CD4+ or CD8+. Lymphocyte > DEAD/CD14-/CD20- > CD3+ > CD4+ or CD8+ > percentage of TNF-α+, IFN-γ+, or CD154+ cells. Arrows indicate sequential steps corresponding to the data in figure S9.

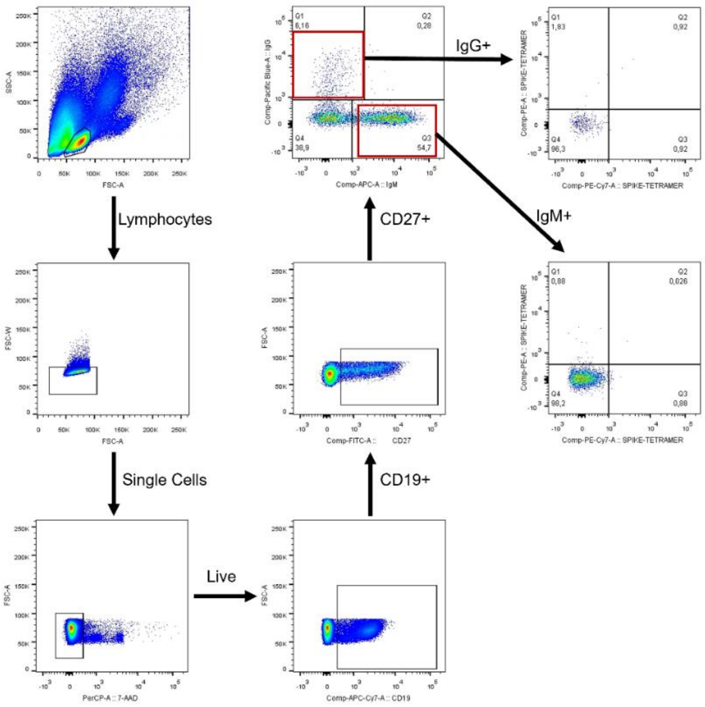

Figure S4. Representative plots (shown as pseudocolor) of the gating strategy used to quantify the percentage of Spike-specific memory B-cells [MBCs] populations. Lymphocyte > Single cells > Live/Dead- > CD19+ > CD27+ > IgG+/IgM- or IgG-/IgM+ > Spike-Protein-PE+/PE-Vio® 770+. Arrows indicate sequential steps corresponding to the data in figure S7 and S8.

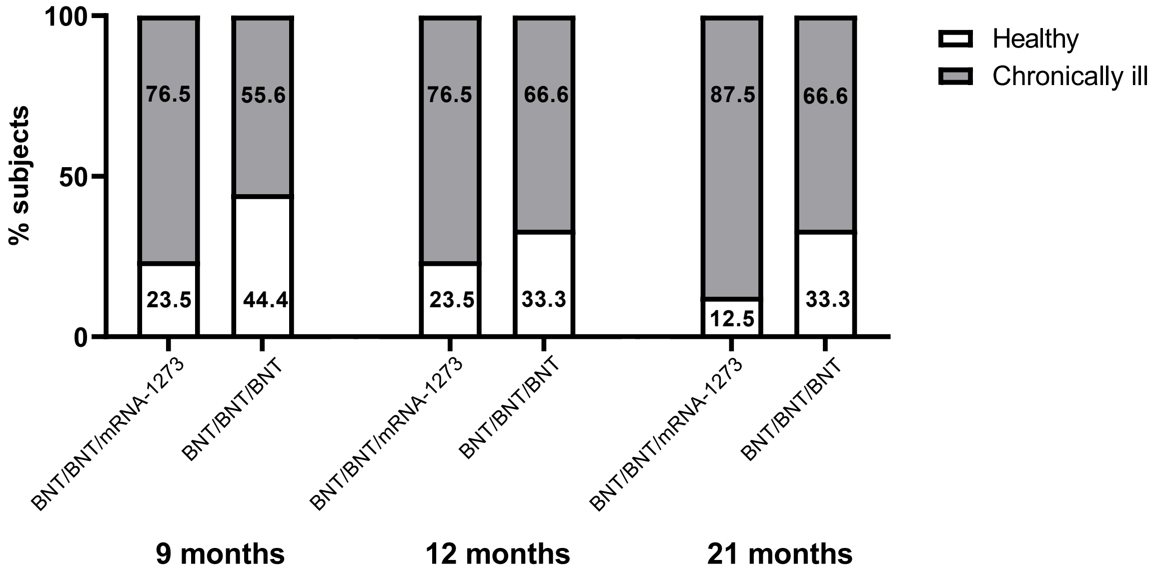

Figure S5. Percentages of individuals with or without chronic health conditions between BNT/BNT/mRNA-1273 and BNT/BNT/BNT vaccinated groups at 9, 12, and 21 months after primary vaccination.

Table S3. Infected [N+] or uninfected [N−] subjects vaccinated with BNT/BNT/mRNA-1273 or BNT/BNT/BNT, grouped according to their chronic condition at month 9, 12, 21.

	9 months				12 months				21 months			
**Pathology**	**BNT/BNT/mRNA-1273** n = 17		**BNT/BNT/BNT** n = 9		**BNT/BNT/mRNA-1273** n = 17		**BNT/BNT/BNT** n = 9		**BNT/BNT/mRNA-1273** n = 8		**BNT/BNT/BNT** n = 3	
	N+ (n = 6)	N− (n = 11)	N+ (n = 2)	N− (n = 7)	N+ (n = 10)	N− (n = 7)	N+ (n = 5)	N− (n = 4)	N+ (n = 6)	N− (n = 2)	N+ (n = 2)	N− (n = 1)
Diabetes	2 (11.8%)	3 (17.6%)	-	1 (11.1%)	3 (17.6%)	2 (11.8%)	-	1 (11.1%)	1 (12.5%)	-	-	-
Cardiovascular disease	-	-	-	2 (22.2%)	-	-	-	2 (11.1%)	-	-	-	-
Respiratory disease	-	2 (11.8%)	-	-	-	1 (5.9%)	-	-	2 (25.0%)	-	-	-
Thrombophilia + MTHFR	1 (5.9%)	-	-	-	1 (5.9%)	-	-	-	-	-	-	-
MTHFR	1 (5.9%)	-	-	-	1 (5.9%)	-	-	-	1 (12.5%)	-	-	-
Autoimmune tyroide	-	1 (5.9%)	-	-	1 (5.9%)	-	-	-	1 (12.5%)	-	-	-
Hypothyroidsm	-	1 (5.9%)	-	-	-	1 (5.9%)	-	-	-	1 (12.5%)	-	-
Hypothyroidsm + MTHFR	-	-	-	-	1 (5.9%)	-	-	-	-	-	-	-
Multiple sclerosis	-	1 (5.9%)	-	-	-	1 (5.9%)	-	-	-	1 (12.5%)	-	-
Dyslipdemia	-	1 (5.9%)	-	-	-	1 (5.9%)	-	-	-	-	-	-
Oncology	-	-	1 (11.1%)	-	-	-	1 (11.1%)	-	-	-	1 (33.3%)	-
K mammary	-	-	1 (11.1%)	-	-	-	1 (11.1%)	-	-	-	-	-
PBC (autoimmune)	-	-	-	-	-	-	1 (11.1%)	-	-	-	1 (33.3%)	-

MethyleneTetrahydrofolate reductase homozygosity mutation [MTHFR]; Primary Biliary Cholangitis (PBC); "-": no chronic pathology. Sample size (n) and relative percentages are reported for each group at each time point.

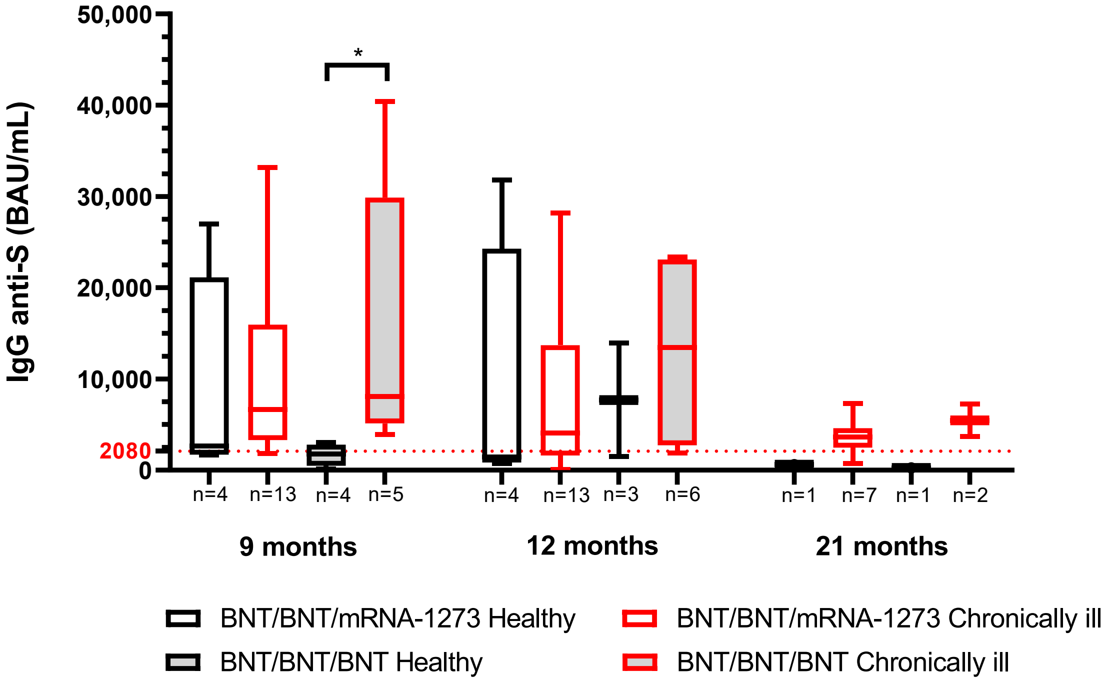

Figure S6. Inter-group comparison of SARS-CoV-2 anti-trimeric Spike protein IgG between two different groups of healthy or chronically ill vaccinated subjects at 9, 12 and 21 months after vaccination. Different numbers of patients reported in the figure. Boxplots display the interquartile range [IQR] and median, with the lowest and highest values represented by whiskers (Tukey-style). Outliers are showed as distinct points. The Kruskal Wallis test with Dunn's post hoc multiple comparison and the Mann-Whitney U test for pairwise comparisons. *$p < 0.05$.

Table S4. Healthy and chronically ill subjects grouped according to SARS-CoV-2 infection (infected [N+] or uninfected [N−]) vaccinated with BNT/BNT/mRNA-1273 or BNT/BNT/BNT, at month 9, 12, 21.

		BNT/BNT/mRNA-1273				BNT/BNT/BNT			
		Healthy		Chronically ill		Healthy		Chronically ill	
		N+	N−	N+	N−	N+	N−	N+	N−
IgG titer (BAU/mL)	9 months Median (IQR)	n = 2/17 (11.8%) 14,340 (1680-27,000)	n = 2/17 (11.8%) 2680 (1780-3580)	n = 3/17 (23.5%) 4570 (2460-15,240)	n = 9/17 (52.9%) 8700 (1814-33,160)	-	n = 4/9 (44.5%) 1780 (138-3040)	n = 2/9 (22.2%) 29,860 (19,320-40,400)	n = 3/9 (33.3%) 6380 (3900-8080)
	12 months Median (IQR)	n = 3/17 (17.6%) 1206 (733-31,800)	n = 1/17 (5.9%) 1700	n = 7/17 (41.2%) 4100 (1530-26,800)	n = 6/17 (35.3%) 3620 (96.2-28,200)	n = 2/9 (22.2%) 10830 (7680-13,980)	n = 1/9 (11.1%) 1540	n = 3/9 (33.3%) 23000 (21,400-23,400)	n = 3/9 (33.3%) 3040 (1884-5480)
	21 months Median (IQR)	n = 1/8 (12.5%) 618	-	n = 5/8 (62.5%) 3640 (2480-4600)	n = 2/8 (25.0%) 4035 (770-7300)	-	n = 1/3 (33.3%) 256	n = 2/3 (66.7%) 5465 (3680-7250)	-

Median and interquartile range (IQR) of SARS-CoV-2 anti-trimeric Spike IgG levels (BAU/mL) at 9, 12 and 21 months post-primary vaccination among the BNT/BNT/mRNA-1273 and BNT/BNT/BNT vaccinated subjects, stratified by infection status and health status (healthy or chronically ill). Sample size (n) and relative percentages are reported for each group at each time point.

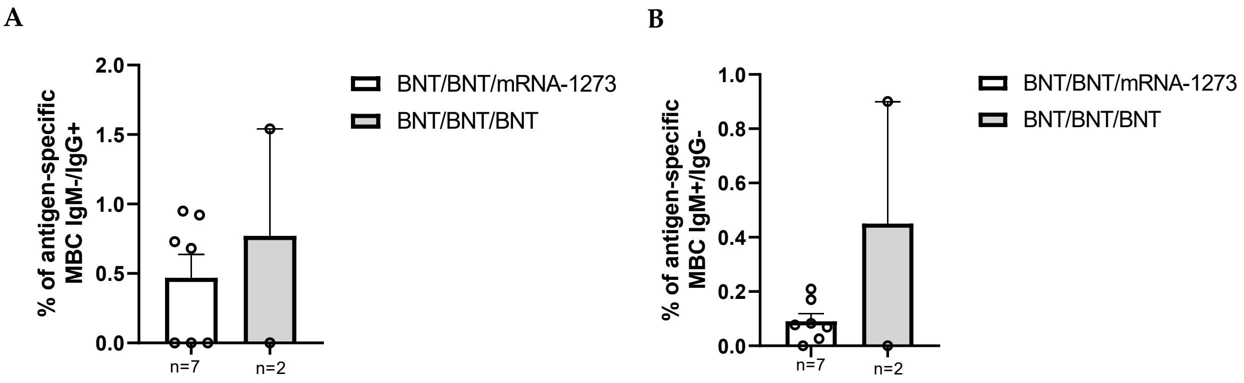

Figure S7. Inter-group comparison of the percentage of Spike-specific memory B-cells [MBCs] between subjects receiving BNT/BNT/mRNA-1273 and BNT/BNT/BNT vaccination schedules at 21 months post-vaccination. A) Percentages of IgM−/IgG+ MBCs. B) Percentages of IgM+/IgG− MBCs. Bars represent the mean with standard error of the mean [SEM]. The number of subjects analyzed in each group is reported below the x-axis. Statistical significance was assessed using the Mann-Whitney U-test.

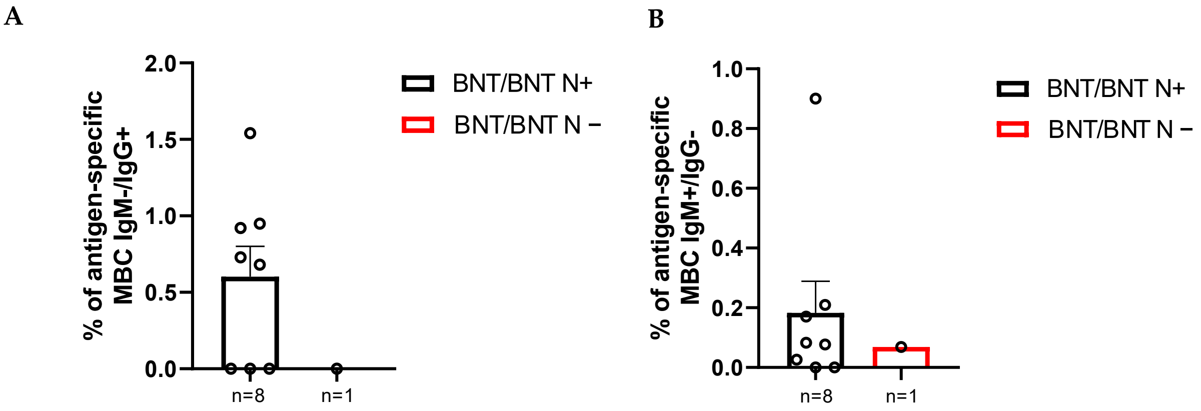

Figure S8. Intra-group comparison of the percentage of Spike-specific memory B-cells [MBCs] between infected/uninfected subjects receiving BNT/BNT vaccination schedules at 21 months post-vaccination. A) Percentages of IgM−/IgG+ MBCs. B) Percentages of IgM+/IgG− MBCs. Bars represent the mean with standard error of the mean [SEM]. The number of subjects analyzed in each group is reported below the x-axis. Statistical significance was assessed using the Mann-Whitney U-test.

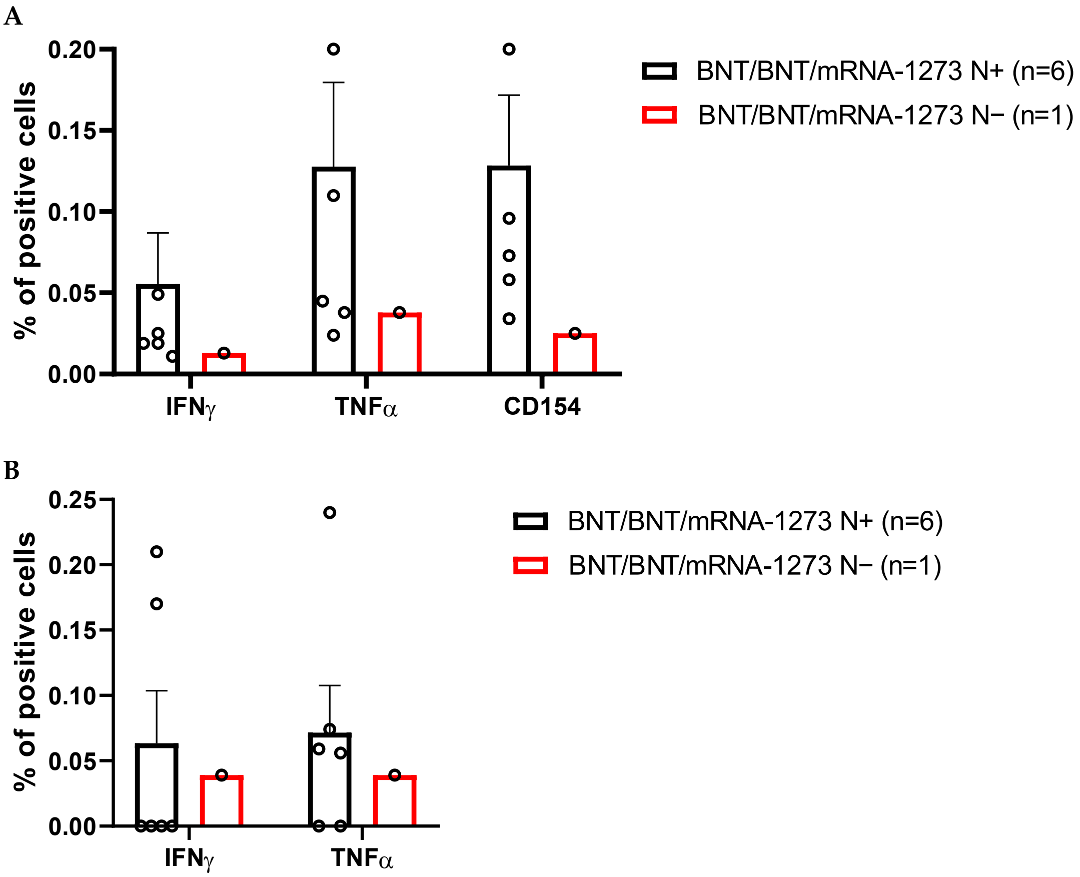

Figure S9. Intra-group comparison of the intracellular cytokine expression in CD4+ and CD8+ T-cells infected/unifected BNT/BNT/mRNA-1273 vaccination groups at 21 months post-vaccination. A) Percentage of CD4+ T-cells positive for IFNγ, TNFα, and CD154 expression. B) Percentage of CD8+ T-cells positive for IFNγ and TNFα expression. Bars represent the mean with standard error of the mean [SEM]. Sample sizes for each group are reported below the x-axis.
